# Fucoxanthin Prevents Long-Term Administration l-DOPA-Induced Neurotoxicity through the ERK/JNK-c-Jun System in 6-OHDA-Lesioned Mice and PC12 Cells

**DOI:** 10.3390/md20040245

**Published:** 2022-03-31

**Authors:** Jingwangwei Liu, Yujia Lu, Min Tang, Fanghao Shao, Dongzi Yang, Shuchang Chen, Ziyi Xu, Leilei Zhai, Juanjuan Chen, Qian Li, Wei Wu, Haimin Chen

**Affiliations:** 1 Collaborative Innovation Center for Zhejiang Marine High-Efficiency and Healthy Aquaculture, Ningbo University, Ningbo 315211, China; 196000502@nbu.edu.cn (J.L.); 196003581@nbu.edu.cn (Y.L.); 196004046@nbu.edu.cn (M.T.); 216000461@nbu.edu.cn (F.S.); 216003481@nbu.edu.cn (D.Y.); 196002221@nbu.edu.cn (S.C.); 216002629@nbu.edu.cn (Z.X.); 2011074058@nbu.edu.cn (L.Z.); chenjuanjuan@nbu.edu.cn (J.C.); 2 State Key Laboratory for Managing Biotic and Chemical Threats to the Quality and Safety of Agro-Products, Ningbo University, Ningbo 315211, China; 3 College of Digital Technology and Engineering, Ningbo University of Finance and Economics, Ningbo 315175, China; liqian@nbufe.edu.cn

**Keywords:** fucoxanthin, antioxidant, Parkinson’s disease, ERK/JNK-c-Jun pathway

## Abstract

As the most abundant marine carotenoid extracted from seaweeds, fucoxanthin is considered to have neuroprotective activity via its excellent antioxidant properties. Oxidative stress is regarded as an important starting factor for neuronal cell loss and necrosis, is one of the causes of Parkinson’s disease (PD), and is considered to be the cause of adverse reactions caused by the current PD commonly used treatment drug levodopa (l-DA). Supplementation with antioxidants early in PD can effectively prevent neurodegeneration and inhibit apoptosis in dopaminergic neurons. At present, the effect of fucoxanthin in improving the adverse effects triggered by long-term l-DA administration in PD patients is unclear. In the present study, we found that fucoxanthin can reduce cytotoxicity and suppress the high concentration of l-DA (200 μM)-mediated cell apoptosis in the 6-OHDA-induced PC12 cells through improving the reduction in mitochondrial membrane potential, suppressing ROS over-expression, and inhibiting active of ERK/JNK-c-Jun system and expression of caspase-3 protein. These results were demonstrated by PD mice with long-term administration of l-DA showing enhanced motor ability after intervention with fucoxanthin. Our data indicate that fucoxanthin may prove useful in the treatment of PD patients with long-term l-DA administration.

## 1. Introduction

Parkinson’s disease (PD) is a progressive neurodegenerative disease, which is characterized by static tremor, bradykinesia, muscle rigidity, mental and cognitive disorders [1]. Based on the 2016 Global Burden of Disease study, PD is the second most common neurodegenerative disease, which is the leading cause of disability worldwide. The current global combined incidence of PD is 0.1–0.2%. However, the incidence rate begins to rise sharply after age 50, rising from 0.4% in the 50–54 age group to 4% in the 80-plus age group [2]. PD, with a mortality rate of 66% within six years, is the third leading cause of death in the elderly after tumors and cardiovascular and cerebrovascular diseases [3]. Therefore, PD has become a major threat to global public health. 

The etiology of PD is related to many factors, such as apoptosis of dopaminergic neurons, excessive oxidative stress, heredity and environmental factors, immune abnormalities, mitochondrial dysfunction, and excitatory toxicity of microglia cells [4]. Recent pathological studies have shown that the significant reduction in dopamine (DA) content in the striatum of patients and the necrosis and loss of dopaminergic neurons in the substantia nigra of the middle brain are important factors inducing PD [5]. However, the current available treatment of PD is mainly given as l-DA or supplementation of DA receptor activators. Although these strategies can relieve PD symptoms to a certain extent, their long-term use will cause adverse reactions, including switch phenomenon, symptom fluctuation, and dyskinesia. Furthermore, studies have shown that excessive concentration of DA in cytoplasm can produce cytotoxicity and lead to the death of DA neurons [6]. This may be due to the fact that DA can be oxidized to hydrogen peroxide and 3, 4-dihydroxyphenylacetaldehyde (DOPAL) by monoamine oxidase in cells, and DOPAL itself is oxidized to produce reactive oxygen species (ROS). DA can also self-oxidize to produce hydrogen peroxide, superoxide anion radical, and hydroxyl radical, which can cause oxidative damage to cells [7]. Therefore, screening and developing neuroprotective drugs to protect or repair DA neurons to slow down or even prevent PD process has become an urgent clinical requirement. 

Although scientists have found some neuroprotective lead compounds, such as PYM-50028, TCH-346, and SR57667, because they are mostly macromolecular proteins, they have poor oral absorption activity and are difficult to cross the blood–brain barrier. The rapid inactivation of enzymes, complex clearance mechanisms, and potential immunogenicity also restrict their access to the central nervous system. It eventually makes almost all these compounds fail after entering the clinic [8]. Recent studies have found that supplementing antioxidants in the early stage of PD can effectively prevent neurodegeneration, improve neuronal function, and inhibit apoptosis in neurons [9,10]. Taghizadeh et al. have reported that pyrroloquinoline quinone neuroprotection might be mediated by the inhibition of oxidative stress and mitochondrial dysfunction [11]. Therefore, achieving the protective effect on neurons through antioxidant methods and effectively preventing the death of neurons, preventing PD process, will be a remission and therapeutic strategy.

**Figure 1:**
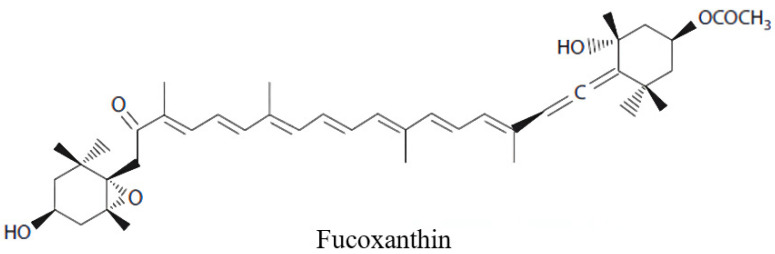
The structure of fucoxanthin.

Fucoxanthin is an oxygen-containing carotenoid, which mainly comes from marine brown algae and microalgae (Figure 1). Due to its unique structure, the presence of the conjugate group functional group, it has a strong antioxidant activity [12]. Moreover, fucoxanthin also has significant anti-inflammatory, neurocytoprotective effects, which are associated with the etiology of PD. However, the safety of fucoxanthin was also affirmed. Even if the intake dose reaches 2000 mg/kg·day, there is still no teratogenic in vivo, and it is considered a safe antioxidant [13]. So far, the molecular target of fucoxanthin, as well as the underlying mechanism of its antioxidant effect, have been preliminarily clarified [14], while the effect of antioxidant properties of fucoxanthin on long-term l-DA treatment in patients with PD remains largely unexplored. 

Therefore, we determined the improved effect of fucoxanthin on apoptosis and mitochondria damage induced by high concentrations of l-DA synergistic 6-OHDA by flow cytometric analysis and fluorescence probe labeling. Furthermore, we analyzed the mechanism of action of fucoxanthin against l-DA together with 6-OHDA-induced cytotoxicity and explored the improved effect of fucoxanthin on locomotion in PD mice with prolonged l-DA administration.

## 2. Results

### 2.1. Fucoxanthin Showed an Improvement in the Cytotoxicity Triggered by High Concentrations of l-DA Coordinated with 6-OHDA

**Figure 2:**
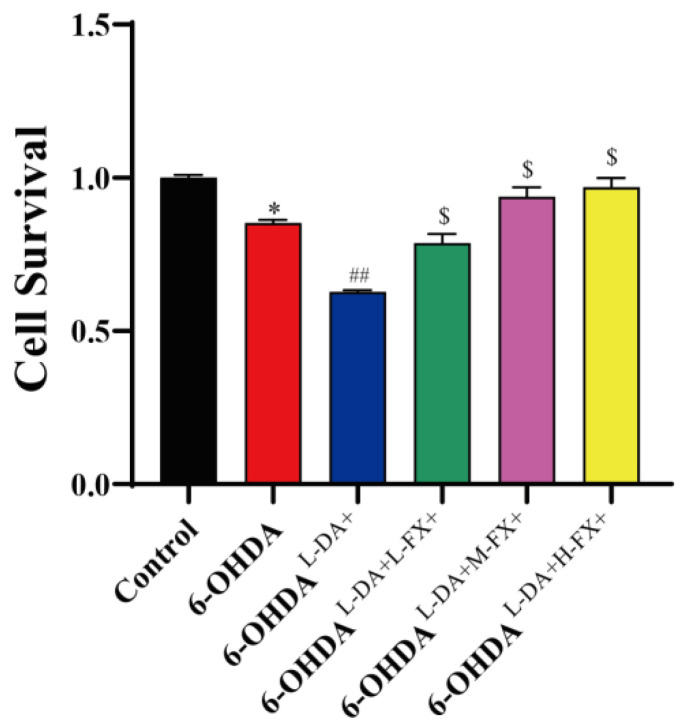
The effect of fucoxanthin (FX) on the survival of high concentrations of l-DA cooperating with 6-OHDA-induced PC12 cells. The data are presented as the mean ± SEM of three independent experiments. * *p* < 0.05 (*n* = 3) compared with the control group, ^##^ *p* < 0.01 (*n* = 3) compared with the 6-OHDA group, and ^$^ *p* < 0.05 (*n* = 3) compared with the 6-OHDA^l-DA+^ group.

To determine whether fucoxanthin improved the cytotoxicity caused by high concentrations of l-DA combined with 6-OHDA, we evaluated cell viability with CCK8 method. As shown in Figure 2, cell survival in 6-OHDA group was significantly reduced by 14.81% compared with control group. Compared with the 6-OHDA group, high concentrations of l-DA combined with 6-OHDA further reduced PC12 cell survival by 26.4% in 6-OHDA^l-DA+^ group (*p* < 0.01). With fucoxanthin pretreatment, the cell survival was increased in a dose-dependent manner. Thereinto, cell survival was significantly increased by 0.54-fold in the 6-OHDA^l-DA+H-FX+^ group (*p* < 0.05) when compared with the 6-OHDA^l-DA+^ group. It demonstrates that fucoxanthin has an ameliorating effect on the cytotoxicity induced by high concentrations of l-DA together with 6-OHDA.

### 2.2. Apoptosis Induced by High Concentrations of l-DA with 6-OHDA Was Improved by Dose-Dependent Fucoxanthin

Next, we examined the effect of fucoxanthin on the apoptosis of l-DA synergistic 6-OHDA-induced PC12 cells and further explored the reasons for its improved cytotoxicity induced by l-DA cooperative 6-OHDA.

**Figure 3:**
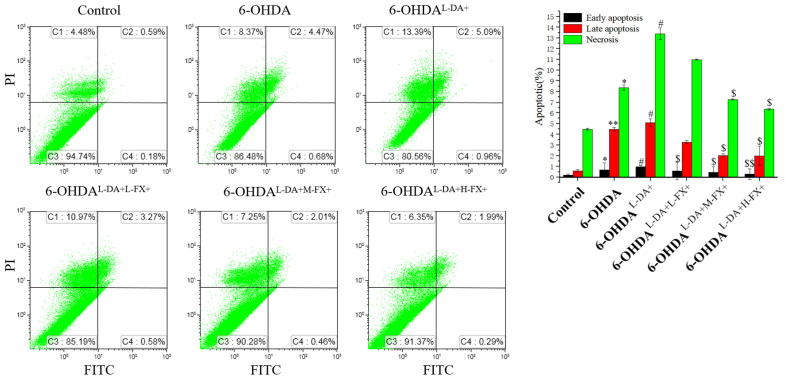
Effects of fucoxanthin (FX) on the apoptosis of PC 12 cells at high concentrations of l-DA combined with 6-OHDA. The treated cells were incubated with Annexin V-FITC and PI and then detected by flow cytometer. The data are presented as the mean ± SEM (*n* = 3). * *p* < 0.05 and ** *p* < 0.01 compared with the control group, ^#^ *p* < 0.05 compared with the 6-OHDA group, ^$^ *p* < 0.05, and ^$$^ *p* < 0.01 compared with the 6-OHDA^l-DA+^ group.

As shown in Figure 3, the rates of early apoptosis, late apoptosis, and necrosis in the 6-OHDA^l-DA+^ group were significantly increased by 0.41, 0.14, 0.60-fold compared with the 6-OHDA group, respectively (*p* < 0.05). Notably, compared with the 6-OHDA^l-DA+^ group, the rates of early apoptosis, late apoptosis, and necrosis significantly decreased by 69.8%, 60.9%, and 52.6%, separately (*p* < 0.05) in the 6-OHDA^l-DA+H-FX+^ group. This result suggests that fucoxanthin downregulates l-DA in concert with the 6-OHDA-induced apoptosis, thus explaining its ameliorating effect on PC12 cytotoxicity. 

### 2.3. Mitochondrial Damage Induced by High Concentrations of l-DA and 6-OHDA Could See a Dose-Dependent Improvement by Fucoxanthin

**Figure 4:**
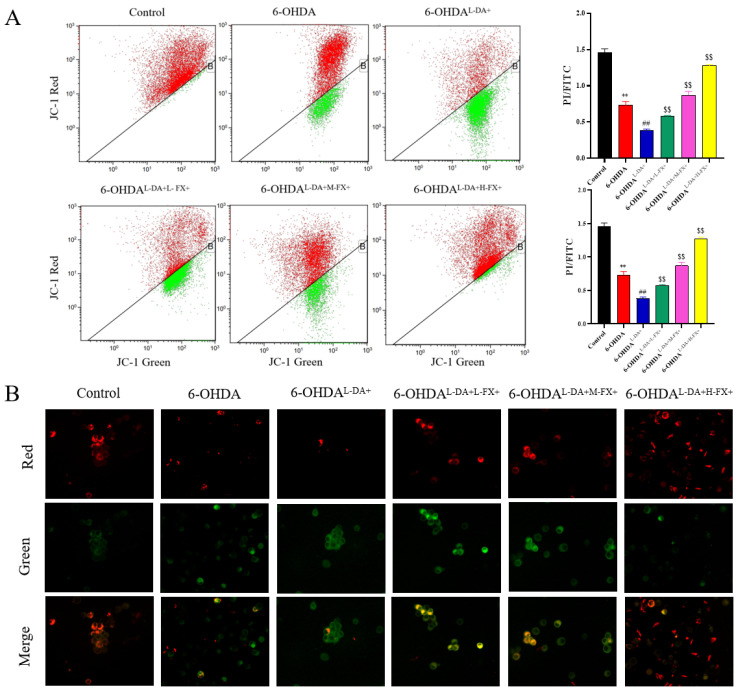
Effect of fucoxanthin (FX) on mitochondrial damage in PC12 cells with high concentrations of l-DA cooperative 6-OHDA damage being observed with the flow cytometry (**A**) and a confocal laser scanning microscope (**B**). The data are presented as the mean ± SEM (*n* = 3). ** *p* < 0.01 compared with the control group, ^##^ *p* < 0.01 compared with the 6-OHDA group, ^$$^ *p* < 0.01 compared with the 6-OHDA^l-DA+^ group.

The earliest event in the apoptotic cascade was the reduction in mitochondrial membrane potential [15]. To further validate the intervention of fucoxanthin on the co-induced apoptosis of 6-OHDA and l-DA, we examined the effects of fucoxanthin on the mitochondrial membrane potential using the JC-1 probe. Compared with the control group, the proportion of cells with damaged mitochondria increased by 11.23-fold in the 6-OHDA group. It shows that 6-OHDA causes severe mitochondrial damage to PC12 cells (Figure 4A). Meanwhile, the ratio of cells with reduced mitochondrial membrane potential in 6-OHDA^l-DA+^ group was further 1.65-fold higher than 6-OHDA group. It is suggested that high concentrations of l-DA would further lead to reduced mitochondrial membrane potential in PC12 cells, that is, increased mitochondrial damage. Notably, the ratio of mitochondrial damaged cells was lower in fucoxanthin intervention groups compared with 6-OHDA^l-DA+^ group, with 6-OHDA^l-DA+H-FX+^ group 90.57% lower than 6-OHDA^l-DA+^ group. Figure 4B shows that red fluorescence decreased in the 6-OHDA group compared to control group, and green fluorescence further increased in 6-OHDA^l-DA+^ group. However, three different concentrations of fucoxanthin intervention groups showed significantly increased red fluorescence compared to the 6-OHDA^l-DA+^ group. These results suggest that fucoxanthin treatment can inhibit the loss or reduction in the triggered mitochondrial membrane potential jointly induced by high concentrations of l-DA and 6-OHDA, contributing to mitochondrial recovery.

### 2.4. The Increase in ROS Levels Caused by the Synergy of High Concentrations of l-DA with 6-OHDA Can Be Inhibited by Fucoxanthin Dose Dependence

**Figure 5:**
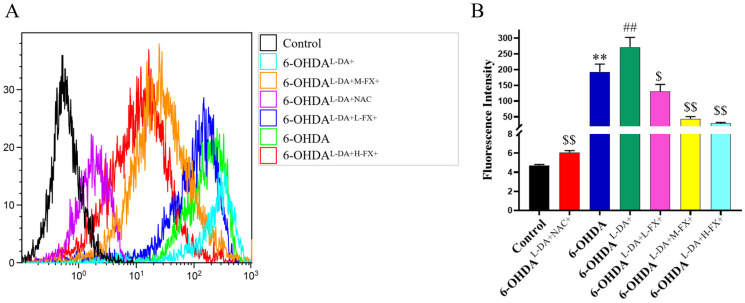
Effect of fucoxanthin (FX) on the changes in ROS levels in PC12 cells caused by the synergy of a high concentration of l-DA with 6-OHDA, cells treated in group were stained with DCFH-DA (10 μM) and examined for ROS levels by flow cytometry (excitation wavelength of 488 nm, emission wavelength of 525 nm) (**A**) and quantitative analysis of the ROS fluorescence intensity of treated PC12 cells by flow cytometry (**B**). These data are presented as the mean ± SEM (*n* = 3) of three experiments. ** *p* < 0.01 compared with the control group, ^##^ *p* < 0.01 compared with the 6-OHDA group, ^$^ *p* < 0.05 and ^$$^ *p* < 0.01 compared with the 6-OHDA^l-DA+^ group.

Several studies have found that the significant reduction in striatal dopamine content and the loss of dopaminergic neurons in the nigra region of the midbrain are important factors in inducing PD [16,17]. Moreover, H_2_O_2_ superoxide anions are produced during dopamine oxidative metabolism, and OH^−^ are generated under the catalysis of Fe^3+^ at the nigra site. The two free radicals of OH promote the oxidation of neuromembrane lipids, resulting in the increase in peroxidation lipids and metabolites malondialdehyde, causing cytotoxicity, resulting in a large number of reactive oxygen species (ROS), secondary cell damage, resulting in apoptosis of dopaminergic neurons [18]. To further explore whether the causes of the changes in apoptosis and necrosis rate are related to ROS, we use oxidation-sensitive DCFH-DA dye, which was employed to determine the intracellular ROS level (Figure 5A). As shown in Figure 5B, 6-OHDA group resulted in extremely significant increase in ROS level compared with that of control group (*p* < 0.01). High concentration of l-DA synergistic with 6-OHDA in the 6-OHDA^l-DA+^ group further resulted in extremely significant increase in ROS levels compared with that of 6-OHDA group (*p* < 0.01). NAC is a hydrogen peroxide scavenger and was designed as a positive control in this experiment. The results showed that supplementing NAC in high concentration of l-DA and 6-OHDA-induced PC12 cells could extremely significantly reduce intracellular ROS compared with that of 6-OHDA^l-DA+^ group (*p* < 0.01). Notably, compared with 6-OHDA^l-DA+^ group, intracellular ROS levels in 6-OHDA^l-DA+M-FX+^ group and 6-OHDA^l-DA+H-FX+^ group were 83.9% and 89.4% lower, respectively (*p* < 0.01). 

### 2.5. Fucoxanthin Downregulated the Expression of Apoptotic Proteins by the Inhibition of the ERK/JNK-c-Jun Pathway in the PC12 Cells

**Figure 6:**
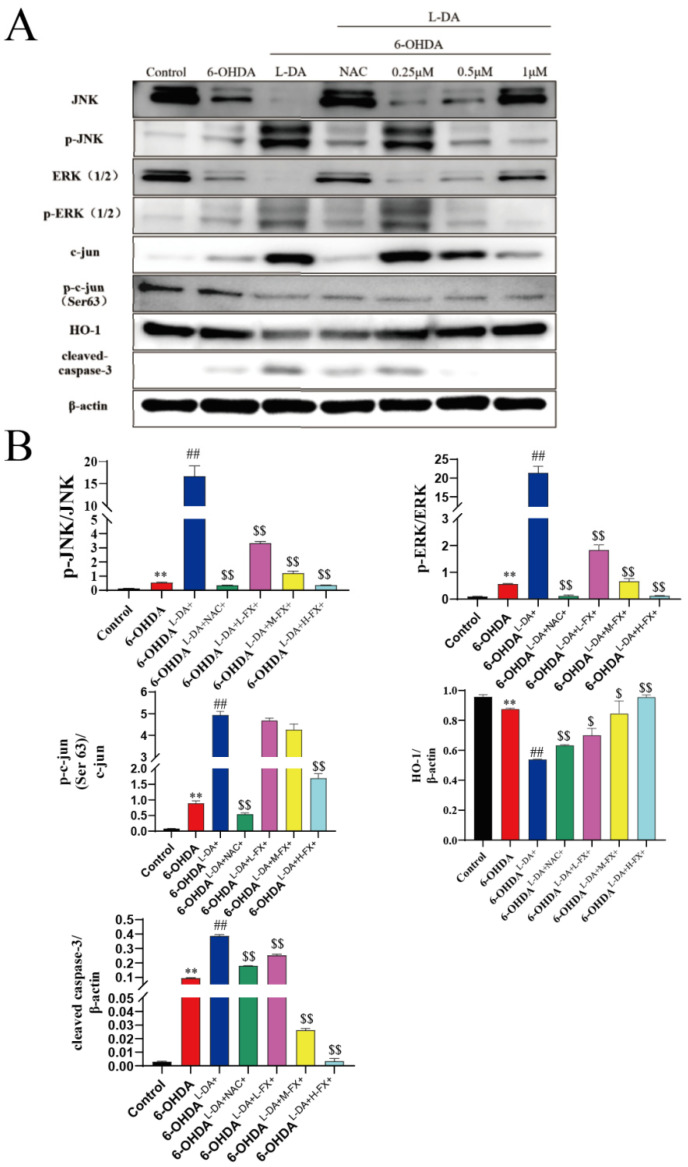
Effect of fucoxanthin (FX) on the ERK/JNK-c-jun pathway in the Parkinsonian model synergistically induced by high concentrations of l-DA and 6-OHDA in PC12 cells. PC12 cells were pretreated with 25 μmol/L PD98059 or SB203580 for 1 h, and then stimulated with 200 μmol/L floridoside for 2 h. Protein expression were determined by western blot (**A**), and the quantification of western blots was analyzed by Fiji software (**B**). The data are presented as the mean ± SEM (*n* = 3) of three experiments. ** *p* < 0.01 compared with the control group, ^##^ *p* < 0.01 compared with the 6-OHDA group, ^$^ *p* < 0.05 and ^$$^ *p* < 0.01 compared with the 6-OHDA^l-DA+^ group.

As shown in Figure 6A, in PC12 cells, 6-OHDA-only treatment significantly increased the phosphorylation of JNK1/2 and ERK1/2 levels in the cells, as well as the phosphorylation of c-Jun at Ser63. Compared with control group, the phosphorylation of JNK1/2, ERK1/2, and c-Jun was increased by 3.09, 4.95, and 9.84 times in 6-OHDA group for PC12 cells, respectively (Figure 6B). On this basis, co-incubation with high concentrations of l-DA furthers significantly increased the phosphorylation of ERK1/2, JNK1/2, and c-Jun. Among them, the phosphorylation of ERK1/2, JNK1/2, and c-Jun in PC12 cells were upregulated by 29.47, 36.86, and 4.55-fold, respectively. At the same time, phosphorylation can be found to significantly inhibit the phosphorylation of ERK1/2, JNK1/2, and c-Jun after treatment with the NAC in the PC12 cells. Upon addition of different concentrations of fucoxanthin intervention, dose-dependent inhibition of ERK1/2, JNK1/2, and c-Jun phosphorylation induced by high concentrations of l-DA co-operative 6-OHDA was observed. In particular, compared with 6-OHDA^l-DA+^ group, phosphorylation of ERK1/2, JNK1/2, and c-Jun in the 6-OHDA^l-DA+H-FX+^ group were down-regulated by 97.8%, 99.4%, and 65.6%, respectively (Figure 6B).

Meanwhile, we found that the 6-OHDA treatment group significantly reduced HO-1 expression, and toxic concentrations of l-DA further reduced the expression of this antioxidase. A dose-dependent increase in HO-1 expression in the fucoxanthin treatment group was noted. The 6-OHDA treatment induced cleaved caspase-3 expression, which was further increased after 24 h of coincubation with high concentrations of l-DA. However, the expression levels of cleaved caspase-3 were suppressed in positive controls treated with NAC in PC12 cells. Furthermore, the dose-dependent fucoxanthin decreased cleaved caspase-3 expression compared to the 6-OHDA^l-DA+^ group. The above results indicate that fucoxanthin downregulates the expression of apoptotic proteins and enhances the expression of antioxidant enzymes by inhibiting the ERK/JNK-c-jun pathway in PC12 cells (Figure 6A,B).

### 2.6. Exercise Ability of PD Mice Induced by High Concentrations of l-DA Could Be Improved by Fucoxanthin

**Figure 7:**
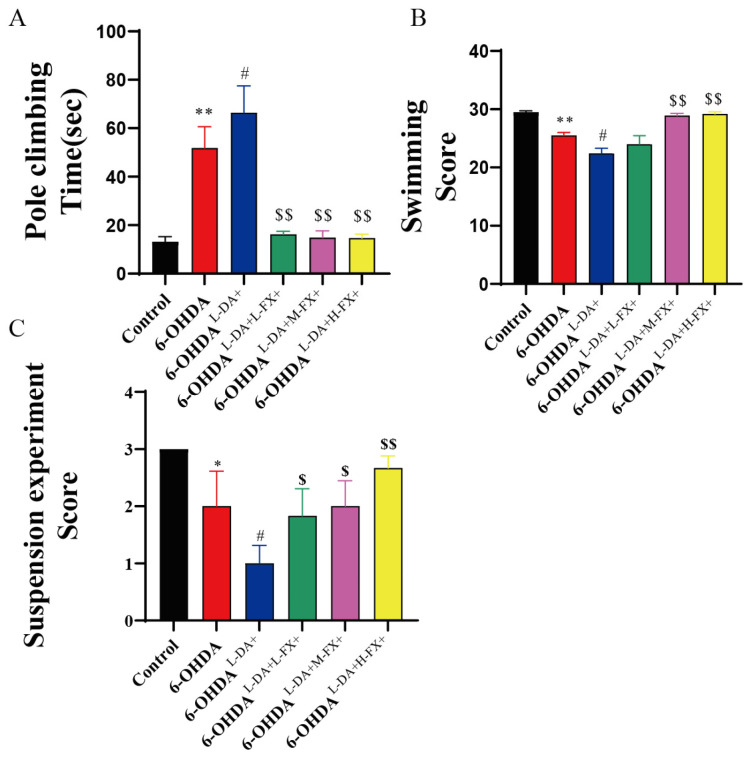
Fucoxanthin (FX) improved the decreased motility induced by high concentrations of l-DA synergy with 6-OHDA. Parkinson mice were treated with behavioral tests, including pole test (**A**), swimming test (**B**), and suspension experiment (**C**) to evaluate their motility ability. The data are presented as the mean ± SEM (*n* = 6) of three experiments. * *p* < 0.05 and ** *p* < 0.01 (*n* = 6) compared with the control group. ^#^ *p* < 0.05 and compared with the 6-OHDA group. ^$^ *p* < 0.05 and ^$$^ *p* < 0.01 compared with the 6-OHDA^l-DA+^ group.

The problem of motor disorder is typical of PD, including bradykinesia, abnormal postural activity, and increased muscle tension [19]. To further assess the intervention effects of different concentrations of fucoxanthin in PD mice treated with high concentrations of l-DA treatment, we detected the mouse motor coordination with the time required for mouse pole climbing. The pole climbing time was significantly increased by 2.94-fold in the 6-OHDA group compared to the control group (*p* < 0.01). However, mice in the 6-OHDA^l-DA+^ group showed a significant 0.28-fold increase compared to 6-OHDA group (*p* < 0.05). Notably, mice treated with different concentrations of fucoxanthin showed a significantly improved performance in the pole test compared to 6-OHDA^l-DA+^ group mice (*p* < 0.01, Figure 7A). Among them, the time required in the 6-OHDA^l-DA+H-FX+^ group decreased by 77.9% compared to the 6-OHDA^l-DA+^ group.

In the swimming test, we assessed the mouse swimming motor behavior. As shown in Figure 7B, the mice in the 6-OHDA group scored significantly lower by 13.6% compared with the control mice (*p* < 0.01). On this basis, the swimming behavior score in 6-OHDA^l-DA+^ group mice significantly decreased by 12.2% compared with 6-OHDA group mice (*p* < 0.05). For the fucoxanthin intervention mice, the motor scores were improved to varying degrees. Notably, mice in the 6-OHDA^l-DA+H-FX+^ group had significantly higher 0.3-fold scores in this test compared to the 6-OHDA^l-DA+^ group (*p* < 0.01, Figure 7B).

We observed a similar phenomenon in the suspension test assessing motor capacity in mice. The PD model mice had lower scores in the suspension tests compared to the control group, and their scores were further decreased after high concentrations of l-DA treatment. Specifically, the scores obtained in the 6-OHDA group were significantly reduced by 33.3% compared to the control mice, while 6-OHDA^l-DA+^ group had a further 50% lower score than 6-OHDA group (*p* < 0.05). Under the intervention of the fucoxanthin, the scores of the groups of mice were improved to varying levels. In particular, the score in the 6-OHDA^l-DA+H-FX+^ group showed a significant 1.67-fold increase compared with the 6-OHDA^l-DA+^ group (*p* < 0.01, Figure 7C).

### 2.7. Fucoxanthin Was Neuroprotective against Mice with PD Models Induced by High Concentrations of l-DA Synergy with 6-OHDA

**Figure 8:**
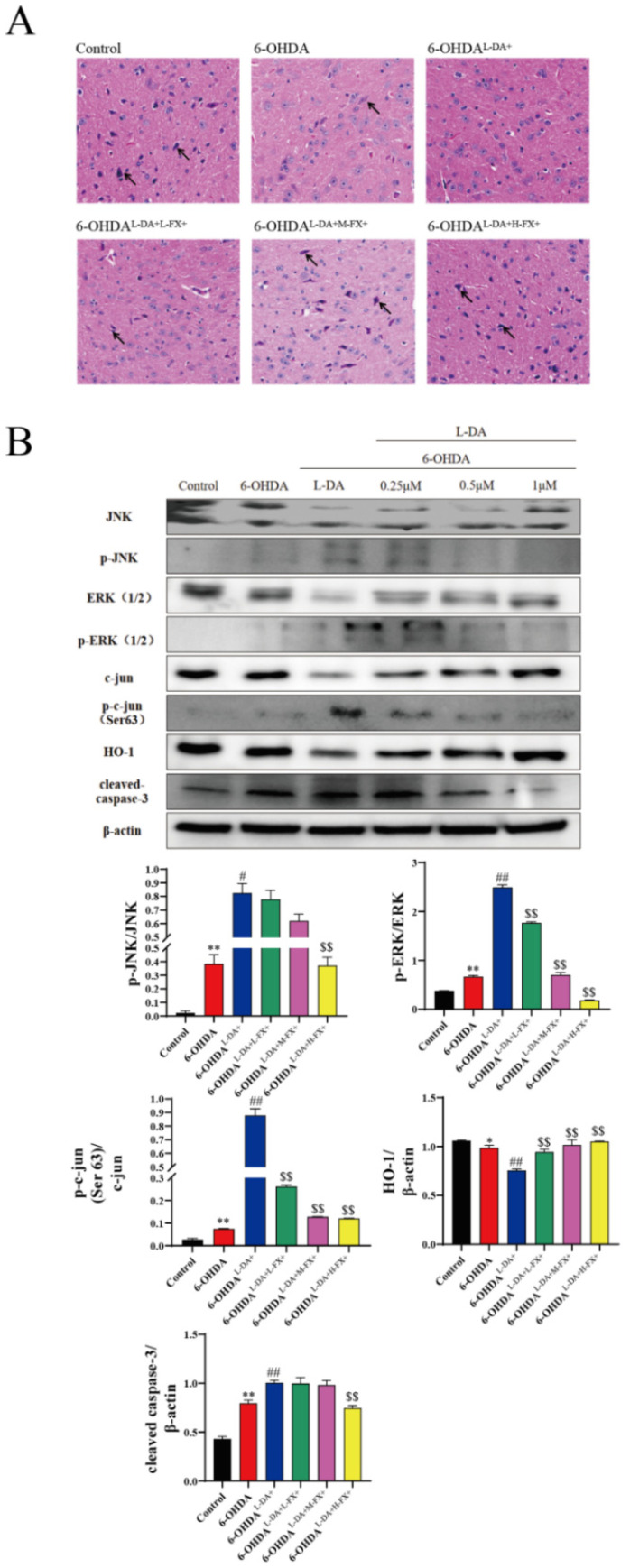
The H&E staining of the mice midbrain substantia nigra. Dopaminergic neuron is indicated with arrowheads (**A**), and effect of fucoxanthin (FX) on the ERK1/2-JNK1/2-caspase3 pathway to the Parkinsonian model synergistically induced by high concentrations of l-DA and 6-OHDA in mice substantia nigra of midbrain (**B**); The data are presented as the mean ± SEM (*n* = 3) of three experiments. * *p* < 0.05 and ** *p* < 0.01 compared with the control group, ^#^ *p* < 0.05 and ^##^ *p* < 0.01 compared with the 6-OHDA group, ^$$^ *p* < 0.01 compared with the 6-OHDA^l-DA+^ group.

The main cause of PD is believed to be the loss of nigra dopaminergic neurons [20]. We sliced and HE stained the nigra region in mice, and the results are shown in Figure 8A. Compared with control group, brain dense dopaminergic neurons of mice were largely absent in the 6-OHDA group, while the number of neurons was further reduced in 6-OHDA^l-DA+^ group. Notably, compared with 6-OHDA^l-DA+^ group, the number of dopaminergic neurons in the nigra region of mice increased with the concentration of fucoxanthin intervention. This suggests that fucoxanthin has a dose-dependent neuroprotective effect on l-DA synergy with 6-OHDA-induced mice.

In mouse midbrain tissue (Figure 8B), treatment of 6-OHDA alone significantly increased the phosphorylation of JNK1/2 and ERK1/2, as well as the phosphorylation of c-Jun at Ser63. The phosphorylation levels of JNK1/2, ERK1/2, and c-Jun were increased significantly by 16.45 times, 0.76 times, and 1.78 times in the 6-OHDA group compared to the control group. Based on this, co-treatment with prolonged l-DA further significantly increased the phosphorylation of ERK1/2, JNK1/2, and c-Jun, increased by 1.15, 2.74, and 10.72-fold, respectively. Following co-addition of 6-OHDA, a dose-dependent inhibition of prolonged l-DA synergy induced ERK1/2, JNK1/2, and c-Jun phosphorylation was observed. In the 6-OHDA^l-DA+H-FX+^ group of mouse brain tissue, the phosphorylation of ERK1/2, JNK1/2, and c-Jun decreased by 55.0%, 92.6%, and 86.3%, respectively.

At the same time, we found that the 6-OHDA treatment group significantly reduced HO-1 expression, while prolonged treatment of l-DA further reduced the expression of this antioxidant enzyme. A dose-dependent increase in HO-1 expression occurred in the fucoxanthin treatment group. The 6-OHDA treatment induced the expression of caspase-3, and it was further increased after a prolonged l-DA co-treatment. Furthermore, a dose-dependent reduction in caspase-3 expression was observed after the addition of the fucoxanthin intervention when compared with the 6-OHDA^l-DA+^ group. The above results indicate that fucoxanthin downregulates the expression of apoptotic proteins and increases the expression of antioxidant enzymes by inhibiting the ERK/JNK-c-Jun pathway in mice.

## 3. Discussion

Oxidative stress leads to neuronal apoptosis via excessive oxidative stress, mitochondrial dysfunction, and finally, cell death. It is considered to be an important starting factor for neuronal cell loss and necrosis, is one of the causes of PD, and is also considered to be the cause of adverse reactions caused by the current PD commonly used treatment drug l-DA [21,22,23]. Therefore, it is a therapeutic and palliative strategy to protect DA neurons and resolutely prevent the death of DA neurons by antioxidation. Some studies have suggested that supplementation with antioxidants early in PD can effectively prevent neurodegeneration and inhibit apoptosis in dopaminergic neurons [9,24]. Fucoxanthin is a natural product of abundant carotenoids and has good antioxidant and neuroprotective activity [25]. Previous reports have confirmed that fucoxanthin could increase the expressions of downstream antioxidant enzymes and decrease the levels of ROS and cell apoptosis in 6-OHDA-exposed cells [14]. 

Mitochondrial dysfunction has been implicated in oxidative stress [26]. Burbulla et al. found that elevated mitochondrial oxidant stress triggered a dopamine-dependent toxic cascade resulting in reduced α-synuclein accumulation, which linked three major pathological features of PD. Furthermore, their data also demonstrated that increased l-DA (the natural precursor of dopamine) contributes to elevated mitochondrial oxidant stress, suggesting a vicious cycle of dopamine and mitochondrial oxidation in human midbrain neurons [9]. In addition, 6-OHDA affects mitochondrial membrane function by suppressing the electron transport chain [26]. Consistent with previous works, our study showed that 6-OHDA triggered a dramatic reduction in mitochondrial membrane potential. In addition, the high concentration of l-DA (200 μM) in 6-OHDA-induced PC12 cells further led to mitochondrial damage and increased apoptosis. However, these results were improved and blocked by FX pretreatment in PC12 cells co-incubated with l-DA and 6-OHDA, and the underlying mechanism might be through reshaping the dynamic balance of ROS and antioxidant levels in vivo and reducing the neurotoxicity induced by dopamine oxidation by increasing the expressions of various antioxidant enzymes. 

The main source of ROS is the mitochondria, and, in turn, ROS also affects mitochondria, leading to a full activation of the caspase activity [27,28]. l-DA-mediated oxidative stress in neuronal or non-neuronal cells affects various signaling pathways, including JNK1/2, ERK1/2, p38 mitogen-activated protein kinase and caspase cascades [29,30]. Keun et al. found that in PC12 cells, high concentrations of l-DA could lead to cell death through oxidative-stress-induced apoptosis, which was mediated by the sustained ERK1/2-JNK1/2-caspase-3 pathway [30]. ERK1/2 phosphorylation increases c-Jun phosphorylation at Ser73 and Ser63, and induces c-Jun expression in PC12 cells [31,32]. JNK1/2 phosphorylation increases c-Jun phosphorylation at Ser73 and Ser63 in sympathetic neuronal cells [33] and also induces c-Jun expression [34]. In these states, c-Jun phosphorylation at Ser73 and Ser63 promotes cell death [31]. In the present study, c-Jun expression was increased by 6-OHDA treatment in PC12 cells, and this expression was further promoted by high concentration of l-DA (200 μM), suggesting that ERK1/2 and JNK1/2 activation mediated by a high concentration of l-DA induces c-Jun phosphorylation. Subsequently, the expression of caspase-3 was promoted, and ultimately, cell death. However, NAC and fucoxanthin partially blocked high concentration of l-DA-induced ERK/JNK-c-Jun system in 6-OHDA-exposed PC12 cells.

The effects of fucoxanthin on long-term l-DOPA-administration-induced dopaminergic neuronal cell loss or apoptosis were investigated in the 6-OHDA-lesioned mice model of PD. The expression of c-Jun is observed in the 6-OHDA-lesioned animal model of PD [35] and is also present in Alzheimer’s disease [36]. The inhibition of JNK1/2 reduces the 1-methyl-4-phenyl-1,2,3,6-tetrahydropyridine-induced dopaminergic neuronal cell death [37]. ERK1/2 activity also decreases in the substantia nigral region of the 6-OHDA-induced rat model of PD [38]. By contrast, ERK1/2 phosphorylation is detected following long-term l-DA administration in the substantia nigral region of 6-OHDA-lesioned rats, which leads to dyskinesia [39]. In the present study, the long-term administration of l-DOPA (30 mg/kg) generated ERK1/2 and JNK1/2 phosphorylation in the 6-OHDA-lesioned midbrains. Under these conditions, c-Jun phosphorylation at Ser63 and caspase-3 expression were increased by l-DA administration (30 mg/kg) for 4 weeks. However, ERK1/2, JNK1/2, c-Jun phosphorylation and caspase-3 were decreased by fucoxanthin treatment in concentration-dependent manner in PC12 cells co-incubated with l-DA and 6-OHDA. These results indicate that long-term administration of l-DA (30 mg/kg) for 4 weeks produces neurotoxicity through caspase-3 and c-Jun expression mediated by the ERK/JNK system. However, these phenomenona may be partially reversed by administering fucoxanthin, improving the exercise ability of PD mice induced by long-term l-DA treatment.

## 4. Materials and Methods

### 4.1. Cell Culture and Fucoxanthin Preparation

Rat pheochromocytoma PC12 cells were purchased from China Center for Type Culture Collection (CTCC, Wuhan, China) and cultured in RPMI1640 medium (Gibco BRL, Grand Island, New York, NY, USA) containing 10% inactivated horse serum (Gibco BRL, Grand Island, New York, NY, USA) supplemented with 10% FBS. PC12 cells were grown in a humidified atmosphere containing 5.0% CO_2_ at 37 °C. Fucoxanthin was provided by Biopurify Co., Ltd. (Chengdu, Sichuan, China), for which the purity of fucoxanthin was 98%, as determined using HPLC detection [40]. Additionally, fucoxanthin was dissolved in dimethyl sulfoxide (DMSO, Sigma, St. Louis, MO, USA) and then diluted to desired concentrations.

### 4.2. Experimental Animals and Experimental Procedure

All C57BL/6 mice were purchased from Beijing Charles River Laboratory Animal Technology Co., Ltd. (Beijing, China) and housed in the animal center of Ningbo University (Ningbo, China). Mice were maintained on a standard rodent chow die and housed in under controlled temperature (21 ± 2 °C) and humidity (50 ± 10%) with a 12 h light and 12 h dark cycle. All experimental procedures were performed in accordance with the National Institutes of Health Guide for the Care and Use of Laboratory Animals, and animal-related experiments were approved by the Ethical Committee of Animal Use and Protection of Ningbo University Health Science Center (protocol code NBU-2021-059 and date of approval 2021-04-08).

PD model mice were obtained by unilateral 6-OHDA injection into the right medial forebrain bundle. After an adaptation period of 1 week, mice were anesthetized by intraperitoneal (i.p.) administration of 1% (*m*/*v*) sodium pentobarbital (60 mg/kg) before being placed in a stereotaxic apparatus (RWD life science, Shenzhen, China). The right lateral brain ventricle (AP = −1.20 mm, ML = −1.20 mm, DV = −4.75 mm, the anterior fontanelle as the origin) was localized with a stereotactic instrument [41]. Additionally, the 6-OHDA (2 μL, 4 mg/mL) was injected through the implanted cannula. Control group mice were administered with an equal volume of saline. Animals were allowed to recover for at least 1 week before post-lesion behavioral testing.

Fucoxanthin (purity > 98%, Biopurify, China) and l-DA were dissolved in olive oil or sterile saline, respectively. Thirty-six C57BL/6 mice (male, 6~8 weeks old, weighing between 18 and 25 g) were randomly divided into six groups (*n* = 6 each group). Control group, 6-OHDA group (6-OHDA surgical operation), 6-OHDA^l^^-DA+^ group (6-OHDA surgical operation and then intraperitoneal injection 30 mg/kg/day l-DA for 14 days), 6-OHDA^l-DA+FX+^ groups (6-OHDA surgical operation, then intraperitoneal injection 30 mg/kg/day l-DA and intragastric administration 50, 100, 200 mg/kg/day fucoxanthin, including 6-OHDA^l-DA+L-FX+^, 6-OHDA^l-DA+M-FX+^, and 6-OHDA^l-DA+H-FX+^ groups) [42]. Fucoxanthin and l-DA were given to each trial once a day for 28 consecutive days. Fucoxanthin was given by oral gavage per morning, and l-DA was given by intraperitoneal injection every afternoon. Mice in the control group were received with identical volume of olive oil by oral gavage and injected with identical volume of saline solution intraperitoneally. Thereafter, behavioral and histologic analyses were performed. All mice underwent euthanasia.

### 4.3. Cell Viability Assay Was Performed by Using Cell Counting Kit-8 (CCK-8)

PC12 cells were divided into control group (equivalent volumes of PBS), 6-OHDA group (225 μM, 48 h), 6-OHDA l-DA+ group (225 μM 6-OHDA treatment for 24 h and then co-incubated with 200 μM l-DOPA for 24 h), 6-OHDA l-DA+FX+ groups (225 μM 6-OHDA treatment for 24 h, then co-incubated with 200 μM l-DOPA and 0.25, 0.5 or 1 μM fucoxanthin for 24 h, including 6-OHDA l-DA+L-FX+, 6-OHDA l-DA+M-FX+, and 6-OHDA l-DA+H-FX+ groups) and seeded in 96-well plates at a density of 1 × 10^4^ cells per well. After treatment, 20 μL of CCK8 solution (Beyotime, Shanghai, China) was added to each well, and the cells were further incubated for 1 h as per manufacturer’s instructions. Then, absorbance values were detected at a wavelength of 450 nm using a multi-detection microplate reader (BioTek, Winooski, VT, USA)

### 4.4. Intracellular ROS Was Measured Using Sensitive Redox Probes (DCFH-DA)

The oxidation-sensitive DCFH-DA dye was employed to determine the intracellular ROS level. The cells were washed with PBS and then incubated with 10 μmol/L of DCFH-DA at 37 °C for 10 min in the incubator. Then, cells were washed twice in ice-cold PBS and analyzed using a Gallios flow cytometer (Beckman Counter, Inc., Brea, CA, USA) at an excitation wavelength of 488 nm and an emission wavelength of 525 nm. NAC (Sigma, 10 mM) was taken as the positive control.

### 4.5. Apoptosis Was Examined by Flow Cytometric Analysis

PC12 cell apoptosis was detected via flow cytometry using the Annexin V/PI Apoptosis Detection kit (Beyotime, Shanghai, China). The treated cells were washed with PBS and suspended in PBS to be 1 × 10^6^ to 5 × 10^6^ cells/mL. Then, cells were resuspended in the ice-cold-binding buffer (BD Biosciences, San Jose, CA, USA) containing FITC-conjugated Annexin V and PI, followed by incubation for 15 min in the dark. The treated cells were then analyzed by a flow cytometer (Beckman Counter, Inc., Brea, CA, USA). Wavelengths for excitation and emission of FITC were 488 and 530 nm, and those for PI were 488 and 630 nm long pass, respectively.

### 4.6. Measurement for Mitochondrial Membrane Potential with Fluorescent Probe JC-1

The early apoptosis of cells was determined by observing the reduction in mitochondrial membrane potential using the JC-1 Mitochondrial membrane Potential Assay Kit (Beyotime, Shanghai, China). The mitochondrial membrane potential was measured by confocal laser microscopy and flow cytometry. Briefly, cells were resuspended in 0.5 mL of RPMI-1640, mixed with 1 mL JC-1 staining solution, and incubated at 37 °C for 20 min. The mitochondrial membrane potential in PC12 cells was measured by flow cytometry (Beckman Counter, Inc., Brea, CA, USA) and a confocal laser scanning microscope (Zeiss LSM 880, Oberkochen, Germany).

### 4.7. Western Blotting

PC12 cells of different treatment groups were washed with PBS and were lysed with cell lysis buffer (Beyotime, Shanghai, China) supplemented with Phosphatase inhibitor cocktail A (Beyotime, Shanghai, China). Samples were then centrifuged and supernatants collected, followed by determination and normalization of protein concentrations. Protein samples were mixed with SDS-PAGE Sample Loading Buffer (Beyotime, Shanghai, China) and boiled for 5 min at 95 °C. 

Mice in different groups were treated with different formulation. Total proteins were extracted from the substantia nigra of each mouse using a total protein extraction kit (Merck Millipore, Billerica, MA, USA) according to the manufacturer’s protocol. Protein samples were mixed with a loading buffer and heated to 95 °C for 5 min. 

Protein samples were run on a 12% SDS–PAGE gel. Afterward, the SDA-PAGE gels were transferred to PVDF membranes (Thermo Fisher Scientific, Dreieich, Germany). After blotting, the PVDF membranes were blocked with 5% non-fat dry milk in TBST (TBS with 0.05% Tween and 0.1% Triton X-100) for 2 h at room temperature. The membranes were washed with TBST and incubated overnight at 4 °C after adding primary antibodies. The primary antibodies used were as follows: β-Actin (1:1000, Cell Signaling Technology, Beverly, MA, USA), cleaved caspase-3 (1:1000, Cell Signaling Technology), HO-1(1:1000, Abcam, MA, USA), p-ERK1/2(1:1000, Cell Signaling Technology), ERK1/2 (1:1000, Cell Signaling Technology), p-JNK1/2 (Thr183/Tyr185) (1:1000, Cell Signaling Technology), JNK1/2 (Thr183/Tyr185) (1:1000, Cell Signaling Technology), p-c-Jun (Ser63) (1:1000, Cell Signaling Technology), c-Jun (1:1000, Cell Signaling Technology). The membranes hybridized to the primary antibody were washed with TBST, followed by incubation for 2 h at room temperature after addition of a horseradish peroxidase-conjugated goat anti-mouse IgG antibody (1:8000, Promega Corporation, WI, USA). After washing with TBST, the chemiluminescent substrate, Immun-StarTM Western CTM Kit (Bio-Rad Laboratories, CA, USA) was added to the membranes, and images were captured with an XRS camera equipped with a Bio-Rad Quantity One imaging system. The quantification of Western blots was analyzed using Fiji software (Image J version 1.48, NIH, Bethesda, MD, USA). 

### 4.8. Behavioral Tests

Mice were acclimated to the behavioral room at least 1 h before testing.

Pole test. The pole test was performed at day 15. A wooden ball with a diameter of 2.5 cm was fixed at the top of a vertical rough-surfaced pole (diameter 10 mm; height 60 cm) glued together with odorless and environmentally friendly glue and placed vertically. Before tests, each mouse was well trained. Mice were placed head upward just below the top of this pole and then allowed to descend. The time until the mouse climbed down to the base of the pole was recorded. This procedure was repeated five times. 

Suspension experiment. For suspension experiment, C57BL/6 mice were placed on a horizontal wire of 1.5 mm in diameter, suspended 25 cm from the ground. The mouse was placed on the wire and only its forepaws were permitted to grasp the wire. The scoring criteria for the suspension experiments are shown in Table A1 (Appendix A).

Swimming test. The coordination of body movement in mice was measured by swimming test. Briefly, mice were placed into a plastic cylinder, 100 cm in diameter and 10 cm deep, filled with water at 22–25 °C and tested in a quiet environment to complete their behavioral scoring. The scoring criteria for the swimming test were shown in Table A2 (Appendix A). 

### 4.9. Hematoxylin and Eosin (H&E) Staining

Briefly, deparaffinized tissue sections were stained with hematoxylin (Sigma-Aldrich, St. Louis, MO, USA) followed by running water. After being exposed to acid alcohol (1% HCl) differentiation solution for 30 s, sections were washed with running water for 6 min. Then, sections were stained with eosin (Sigma-Aldrich, St. Louis, MO, USA) for 2 min, followed by a 6 min wash with running water. Graded ethanol and xylene were used to dehydrate sections and neutral balsam (Sinopharm Chemical Reagent Co., Ltd., Shanghai, China) was used to mount it.

### 4.10. Statistical Analysis

Experimental data were assessed using the SPSS 16.0 software (SPSS Inc., Chicago, IL, USA). Data were expressed as means ± SEM. Statistical significance was determined by one-way ANOVA. Mean comparison analyses were performed with Tukey’s HSD post hoc test. Statistical significance was considered to be significant (*p* < 0.05) and extremely significant (*p* < 0.01).

## 5. Conclusions

The present results showed that fucoxanthin provided neuroprotection against the apoptosis of PC12 cells and the loss of nigra dopaminergic neurons and behavioral impairment caused by 6-OHDA synergies with long-term l-DA administration. Fucoxanthin could suppress the high concentration of l-DA-mediated neuronal death in the 6-OHDA induced PC12 cells and mice model of PD through improving reduction in mitochondrial membrane potential, suppressing ROS over-expression, and inhibiting activation of ERK/JNK-c-Jun system. These findings suggest that fucoxanthin has pluripharmacological properties in the protection against PD, including enhancing antioxidant activity and inhibiting mitochondria-mediated apoptosis. 

## Data Availability

All data needed to evaluate the conclusions in the paper are present in the paper.

## Appendix A

**Table A1:** The suspension ability test scoring system for Parkinson model mouse behavioral experiments.

Score	Training Situation	Number of Replications	Suspension Behavior of the Mice
0	Each mouse was trained 3 days before surgery	Each mouse tested three times	Fell immediately
1			Two hindlimbs were not able to grasp the wire
2			Only one hindlimb was able to grasp the wire
3			Two hindlimbs were able to grasp the wire

**Table A2:** The swimming exercise behavioral scoring system for Parkinson model mouse behavioral experiments.

Score	Testing Time	Number of Replications	Swimming Behavior of the Mice
10–15	1 min	Each mouse tested three times	Hindlimbs occasionally swim and floating on one side of the tank
15–20			swimming by chance
20–25			Floating time accounts for more than half of the time
25–30			Swimming most of the time, floating by chance
30			Keep swimming for a minute
